# *Sumac* Silver Novel Biodegradable Nano Composite for Bio-Medical Application: Antibacterial Activity

**DOI:** 10.3390/molecules200712946

**Published:** 2015-07-17

**Authors:** Parisa Ghorbani, Mozhgan Soltani, Masoud Homayouni-Tabrizi, Farideh Namvar, Susan Azizi, Rosfarizan Mohammad, Amin Boroumand Moghaddam

**Affiliations:** 1 Department of Biology, Science and Research Branch, Islamic Azad University, Tehran 14911, Iran; E-Mail: prspink91@yahoo.com; 2 Department of Biology, Mashhad Branch, Islamic Azad University, Mashhad 917568, Iran; E-Mail: Soltani_m4@yahoo.com; 3 Department of Biochemistry and Biophysics, Mashhad Branch, Islamic Azad University, Mashhad 917568, Iran; E-Mail: mhomayouni6@gmail.com; 4 Institute of Tropical Forestry and Forest Products (INTROP), Universiti Putra Malaysia, UPM Serdang, Selangor 43400, Malaysia; 5 Research Center for Animal Development Applied Biology, Mashhad Branch, Islamic Azad University, Mashhad 917568, Iran; 6 Department of Bioprocess Technology, Faculty of Biotechnology and Biomolecular Sciences, Universiti Putra Malaysia, UPM Serdang, Selangor 43400, Malaysia; E-Mails: azisusan@gmail.com (S.A.); amin.broomandm@yahoo.com (A.B.M.)

**Keywords:** silver nanocomposite, *sumac*, antibacterial activity, green synthesis

## Abstract

The development of reliable and ecofriendly approaches for the production of nanomaterials is a significant aspect of nanotechnology nowadays. One of the most important methods, which shows enormous potential, is based on the green synthesis of nanoparticles using plant extract. In this paper, we aimed to develop a rapid, environmentally friendly process for the synthesis silver nanoparticles using aqueous extract of *sumac*. The bioactive compounds of *sumac* extract seem to play a role in the synthesis and capping of silver nanoparticles. Structural, morphological and optical properties of the nanoparticles were characterized using FTIR, XRD, FESEM and UV-Vis spectroscopy. The formation of Ag-NP was immediate within 10 min and confirmed with an absorbance band centered at 438 nm. The mean particle size for the green synthesized silver nanoparticles is 19.81 ± 3.67 nm and is fairly stable with a zeta potential value of −32.9 mV. The bio-formed Ag-NPs were effective against *E. coli* with a maximum inhibition zone of 14.3 ± 0.32 mm.

## 1. Introduction

Recently, there has been much research interest in nanomaterials. Nanomaterials are produced through physical, chemical and biological process. So far, chemical and physical methods have been applied to synthesize nanoparticles of various sizes and morphologies. However, these processes are toxic and costly, respectively. Thus, researchers are focusing their efforts on replacing these methods with green and economical biological procedures which happen at ambient conditions and produce nanoparticles which are extremely stable under strong electrolytic and pH conditions for therapeutic application [1]. The living organisms which have shown potential for the production of various nanoparticles are plants [2], alga [3], and different microorganisms, for example, fungi [4], bacteria [5] and actinomycetes [6]. Among the biological system of synthesis of nanoparticles, the plant-mediated system is gaining significance due to its simplicity, eco-friendliness and wide medicinal effects [7]. The biomolecule present in aqueous extract of the plant has the potential to be absorbed on the surface of nanoparticles during particle formation procedures, which eventually leads to the occurrence of succeeding different surface effects during their application. Therefore, study of the different plant sources which have different extract compositions as the mediate for nanoparticles production could be interesting.

*Sumac* (Rhuscoriaria L) belongs to the Anacardiaceae family and grows in Mediterranean countries, North Africa, south Europe, Iran and Afghanistan. The fruits of *sumac* contain flavonoids, phenolic acids, hydrolysable tannins, anthocyans and organic acids such as malic, citric and tartaric acids [8,9]. *Sumac* extracts have been found to have antimicrobial, antioxidant and hypoglycemic activities [9]. The chemical compositions of *sumac* have potential for silver reduction and modify the surface structural property of particles.

Silver nanoparticles (Ag-NPs) are among the most widely studied in metallic nanoparticles that possess unique physicochemical properties [10]. Ag-NPs are widely investigated due to their broad range of applications as antibacterial, catalyst, anti-HIVactivity, controlling plant pathogens and as a biosensor [10,11,12,13]. In recent years, Ag-NPs have merited substantial attention for the production of a new class of antimicrobials [14] opening up a new approach to contest a broad range of bacterial pathogens [15]. Some studies have reported that the use of green gentle materials such as seed aqueous extract (*Pistacia atlantica*) [16], bacteria [17], and alga [18] for the production of silver nanoparticles offers many benefits, including eco friendliness and compatibility for biomedical applications, where toxic chemicals are not used for the production process. The green production of intrinsically safer Ag-NPs depends upon the the basic requirements of green chemistry: the solvent medium, the gentle reducing agent and the safe capping agent [19]. For this purpose, we used aqueous extract of *sumac* to synthesize benign Ag-NPs as it fulfills the above-mentioned requirements. The history, chemical and biomedical properties of *sumac* have been well reviewed and documented elsewhere, but the ability of biomolecules present in *sumac* for nanoparticle synthesis is unexplored. Hence, the present study was designed to synthesize and characterize biosynthesized silver nanoparticles by using aqueous extract of *sumac*. In addition, we evaluated the antibacterial activity of the bioformed nanoparticles against a Gram-negative bacterium, *E. coli*, an important pathogen of the intestine of warm-blooded organisms.

## 2. Results and Discussion

Currently, green synthesis of nanoparticles through plant extracts is under exploitation. The growth of biologically inspired experimental procedures for the synthesis of nanoparticles is developing into a significant aspect of nanotechnology [20]. The present study deals with the synthesis of silver nanoparticles using water extract of *sumac* and aqueous Ag^+^ ions. The method appears to be an environmentally simple and cost effective alternative to conventional approaches of synthesis silver nanoparticles. The results along with their discussion are given below.

**Figure 1:**
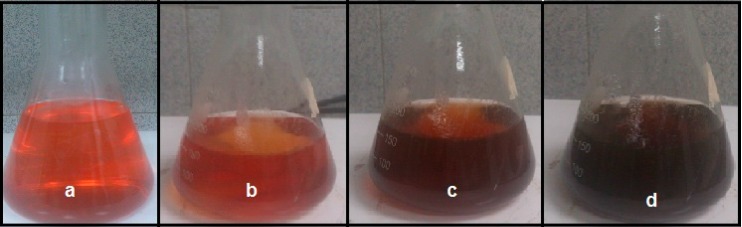
Synthesis of silver nanoparticles (Ag-NPs) using aqueous extract of *sumac*: (**a**) aqueous extract of *sumac*; (**b**) initial point of time; (**c**) mid-point of time and (**d**) final point of time.

**Figure 2:**
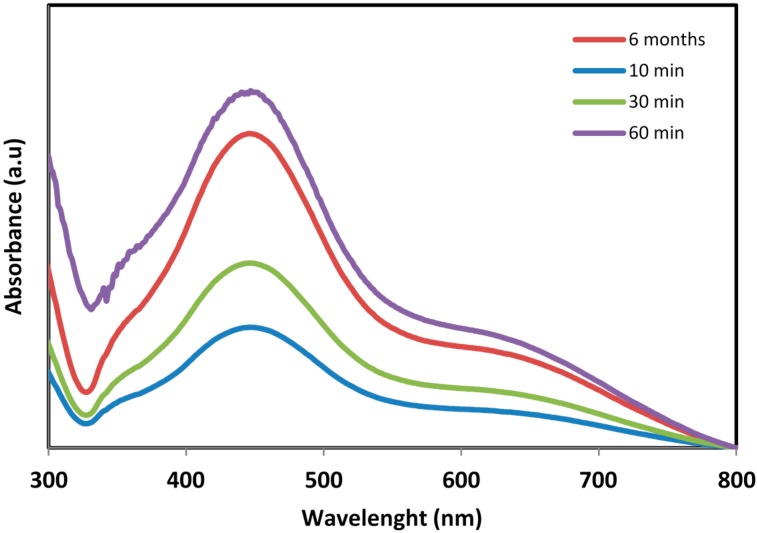
UV-visible spectra of bio-synthesized Ag-NPs.

In this study, the formation of Ag-NPs in Ag^+^/*sumac* solution was observed through visual assessment. Figure 1 clearly shows that the color of solution was changed from agate red color to light brown within 10 min and then to dark brown after 1 h, which indicated the completed synthesis of Ag-NPs [21]. The appearance of light/dark brown color was due to excitation of Surface Plasmon vibrations, due to the combined vibration of electrons of the silver nanoparticles in resonance with the light wave [22,23]. The bio-formed silver nanoparticles showed an absorption maximum at 438 nm in the visible region (Figure 2) with light-brown or dark-brown color [24]. Because of the excitation of the plasmon resonances of inter band transitions, some metallic nanoparticle dispersions display unique bands/peaks [25]. The wideness of the peak is good evidence of the nanoparticle size [26,27]. It can be observed that the absorption gradually increases in intensity as a function of time of reaction, indicating an increase in the number of formed Ag-NPs in the solution. Furthermore, the SPR band centered at 434 nm after 6 months indicating that these particles were stable for more than 6 months when kept at room temperature.

**Figure 3:**
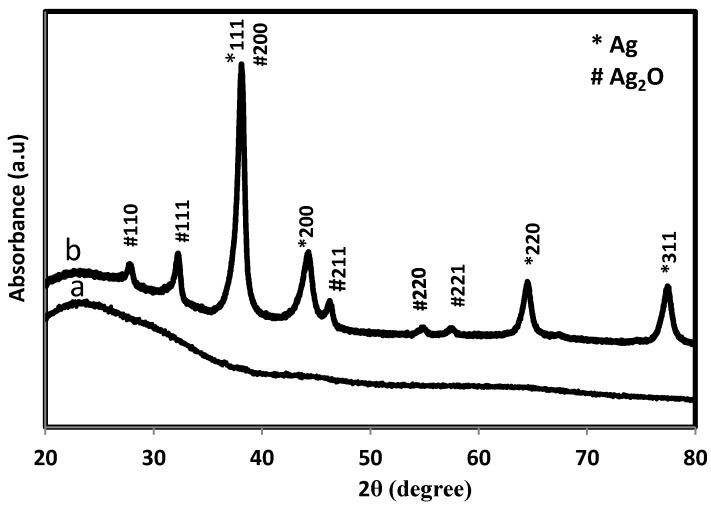
XRD pattern of *Sumac* (**a**) and bio-formed NPs (**b**).

XRD patterns of the *sumac* and bio-formed silver nanoparticles are shown in Figure 3. In case of silver containing sample, The XRD peaks at 38.23°, 44.27°, 64.49° and 77.56° can be indexed to the (111), (200), (220) and (311) Bragg’s reflections of face center cubic (fcc) structure of metallic silver respectively similar to Joint Committee on Powder Diffraction Standards (JCPDS) file no: ICDD-PDF2, revealing that biosynthesized Ag-NPs are of crystalline silver. On the other hand, the peaks at 27.90°, 32.30°, 38.23°, 46.17°, 54.82°, 57.02° can be assigned to the (110), (111), (200), (211), (220) and (221) Bragg’s reflections peaks corroborate with the standard Ag_2_O (JCPDS 01-075-1532). Previous studies show that flavonoid contents of the plant extract provide the electron to reduction metal ions to nano zero valent metallic particles [28], but the clear mechanism resulting in the formation of silver oxide nanoparticles by plant extract has not been reported. There is a possibility that a small number of nano zero valent silver nanoparticles were converted into silver oxide by some oxidizing agent molecules present in extract. However more studies are needed in this way to verify the exact mechanism involved in the formation of silver oxide nanoparticles.

**Figure 4:**
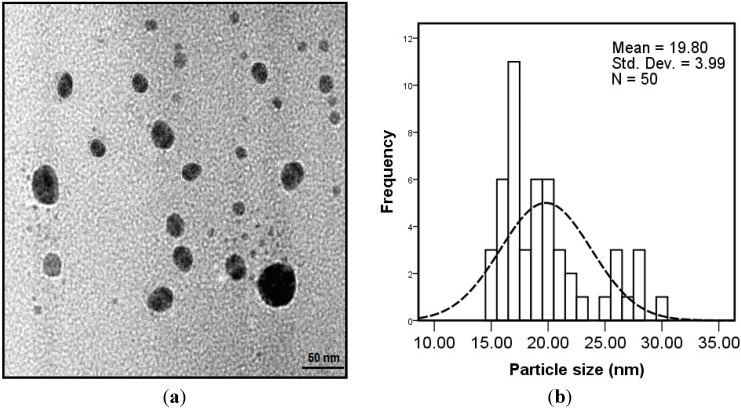
TEM image (**a**) and particle size distributions (**b**) of bio-synthesized nanoparticles.

The TEM image Figure 4a and particle size distribution graph Figure 4b show that the Ag-NPs that formed were well dispersed with spherical structures and particle size ranging from 15 to 30 nm with some deviations.

**Figure 5:**
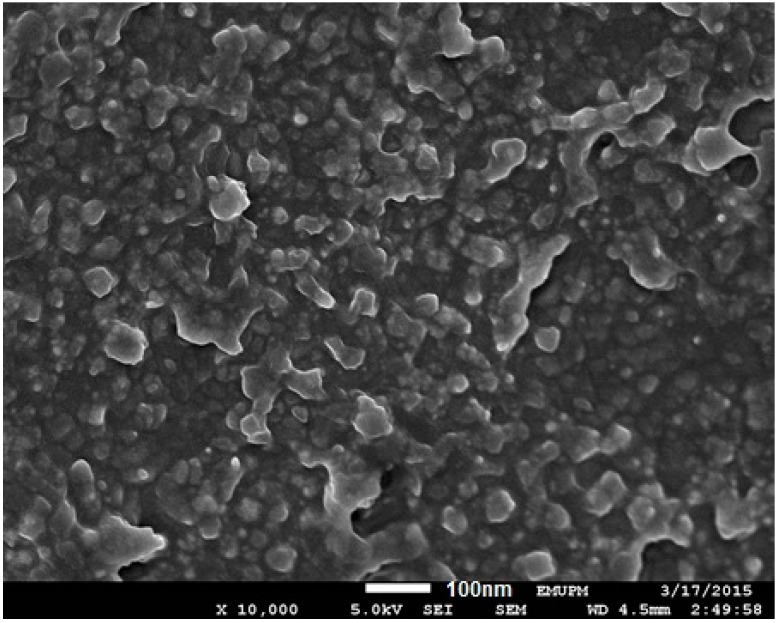
Field emission scanning electron microscopy (FESEM) image of Ag-NPs synthesized using *sumac* extract.

The morphology and structure of the NPs were further investigated by FESEM. The FESEM image of prepared Ag-NPs was showed in Figure 5. The Ag-NPs are quasi-spherical in shape and coated with biomolecules present in *sumac* extract with a white boundary on the surfaces of nanoparticles. The results are consistent with the concept that the Ag nanoparticles are firmly coated by bio-compounds present in *sumac* and this process inhibits further aggregation or agglomeration between the final nanoparticles.

**Figure 6:**
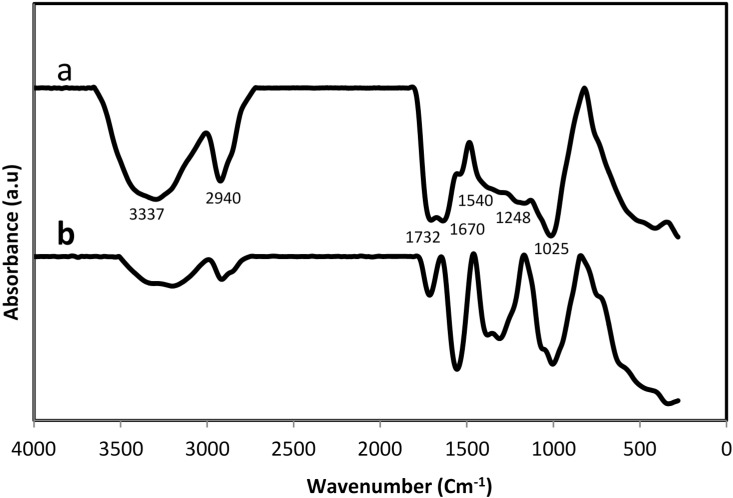
FTIR spectra of *sumac* before (**a**) and after (**b**) synthesis of Ag-NPs.

FT-IR analyses were performed to identify the potential bio-compounds or molecular components in *sumac* responsible for reduction and efficient capping of the bio-formed silver nanoparticles. The FTIR spectrum is shown in Figure 6a,b. The observed peaks were more characteristic of flavonoids and tannins that are very abundant in *sumac* extract. The 3337 cm^−1^ absorption band is accredited to –OH stretching vibration, the band at 2940 cm^−1^ is due to –CH and –CH_2_ vibration of aliphatic hydrocarbon. The band at 1732 cm^−1^ corresponds to carbonyl group (C=O), the bands at 1670 and 1540 cm^−1^ are assigned to aromatic ring stretching vibration. The bands at 1248 and 1025 cm^−1^ can be assigned to the ethereal C–O asymmetric stretching vibration arising from the pyran-derived ring structure of condensed tannins [29].

The presence of flavanoid, tannins and other phenol compounds in the extract could be responsible for the reduction of silver ions and synthesis of Ag-NPs. Moreover, the FT-IR of extract after formation of Ag-NPs shows demonstrative differences in the shape and position of signals signifying the involvement of functional groups of biomolecules for production and capping of metallic nanoparticles. Polyphenols could be adsorbed on the surface of metal nanoparticles, probably by interaction through π electrons interaction in the absence of other strong ligating agents. In fact the π electrons of carbonyl group (C=O) from C ring of flavonoids or from carboxyl groups of tannin in a Red/Ox system can transfer to the free orbital of metal ion and convert that to the free metal [28]. This is confirmed by a reduction in intensity of the peak at 1732 cm^−1^. It is also possible that the phenolic compounds play an important role in the reduction of metal ions by oxidation of hydroxyl groups (3337 cm^−1^) to aldehyde [30].

**Figure 7:**
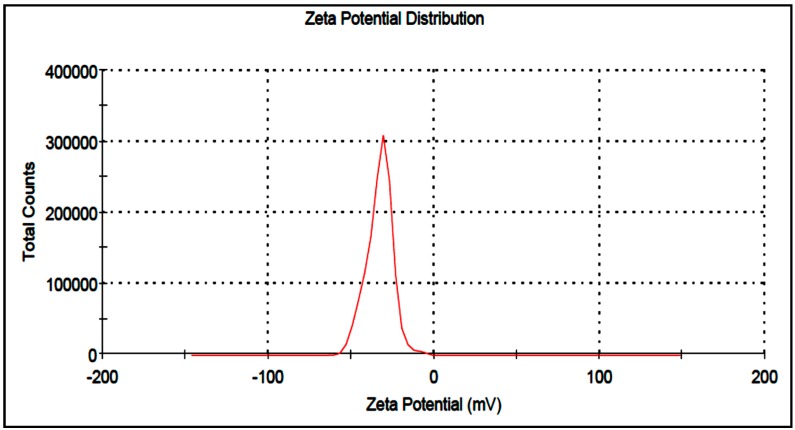
Zeta potential of Ag-NPs synthesized using *sumac*.

As shown in Figure 7, the zeta potential value of silver nanoparticles are −32.9 mV, indicating the particles are warped with anionic bio-molecules and highly stable. The higher zeta-potential value is an important parameter to continue the stability of suspension because it will cause a repulsive force and retain the nanoparticles away from each other, which results in a high stability of suspension.

### 2.1. Antimicrobial Activity

**Figure 8:**
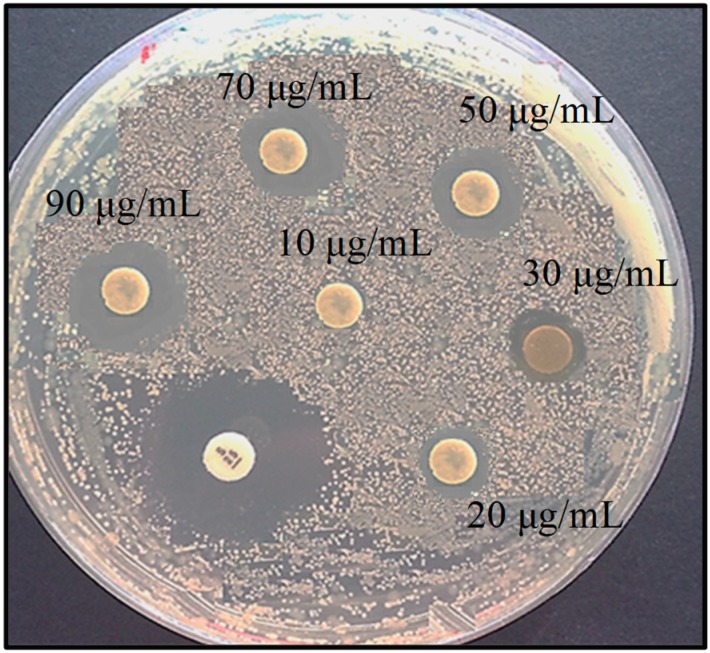
Inhibition zone of the bio-formed Ag-NPs solution against *E. coli* pathogen.

In this study the antimicrobial activity of biosynthesized Ag-NPs with different concentrations against Gram negative bacteria *E. coli* was determined in terms of the inhibition zone. In this analysis, the various concentrations of Ag-NPs displayed antimicrobial activity against *E. coli* (Table 1, Figure 8). The mean of three replicates of the diameter of the zone of inhibition for each concentration were determined to be about 9.1 ± 0.13, 10.9 ± 0.26, 11.8 ± 0.41, 13.3 ± 0.12, 14.3 ± 0.32 mm, respectively, for 20 μg/mL, 30 μg/mL, 50 μg/mL, 70 μg/mL, and 90 μg/mL. The findings propose that the Ag-NPs act as an effective antibacterial agent. As seen, with the increase of Ag-NPs concentration, the diameter of the inhibition zone increased gradually. This effect may have resulted from more accumulation of nanoparticles on the cell surface which interact with the microbial membrane to cause structural change and permeability, and finally bacteria cells death [31]. The antimicrobial activity is extremely influenced by the size, shape and concentration of nanoparticles [32,33] and in a study using *E. coli* [34] established that Ag-NPs accumulation on the membrane cell makes gaps in the entirety of the bilayer which predisposes it to a penetrability increase and finally bacterial cell death [31].

**Table 1:** Mean inhibition zone (mm) of biosynthesized Ag-NPs against *E.coli* pathogen.

Concentration (μg/mL)	Inhibition Zone (mm)
10	-
20	8.5 ± 0.53
30	10.2 ± 0.46
50	11.8 ± 0.41
70	13.3 ± 0.12
90	14.3 ± 0.32
Positive control	17.2 ± 0.38

### 2.2. Cell Cytotoxicity

**Figure 9:**
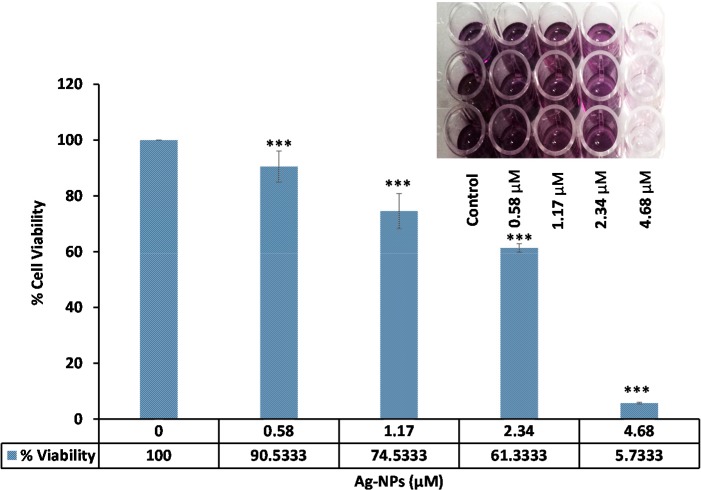
Cytotoxic effect of biosynthesized Ag-NPs on the growth of HepG2. A dose-dependent reduction of cell viability was observed at 570 nm. *** *p* < 0.001.

The MTT assay was employed to assess the cytotoxic potential of biosynthesized Ag-NPs. Figure 9 shows that increasing concentrations Ag-NPs reduce HepG2 viability, and the reduction of color at 570 nm in comparison to untreated cells revealed the cytotoxicity of the NPs.

## 3. Experimental Section

Ground *sumac* fruit with red color was bought in bulk from local market in Iran. AgNO_3_ (99.98%) was used as silver precursor and it was supplied from Merck (Darmstadt, Germany). All the solutions were prepared with deionized water.

### 3.1. Preparation of Sumac Extracts

*Sumac* sample (1 g) was dispersed in 100 mL distilled water by magnetic stirring and heated at 100 °C for 30 min. The extract was filtered through mesh, followed by Millipore filter (0.2 μm), and stored at −20 °C before use.

### 3.2. Biosynthesis of Ag-NPs

The aqueous solution (0.5 mM) of AgNO_3_ was mixed with 50 mL of the aqueous extract of *sumac* for 60 min under continuous stirring at 35 °C and then allowed to stand at room temperature for another 2 h. The dark brown solid product was collected through centrifugation at 10,000 rpm for 10 min and careful washing with distilled water. The final products were obtained by drying at 45 °C.

### 3.3. Characterization of Ag-NPs

Phase purity was determined by X-ray diffraction (XRD) analysis recorded by diffractometer (XPERT-PRO) with nickel-filtered Cu (λ = 1.542 Å) operated at 40 kv and 30 mA at room temperature in the range from 10° to 80°. The chemical structure of powder samples were studied by using a FTIR spectrometer (Perkin-Elmer 1725X, Waltham, MA, USA at wavenumber range from 400 to 4000 cm^−1^. The disk containing 1 mg of the sample was scanned within a wave number range of 400 to 4000 cm^−1^. Transmission electron microscopy (TEM) observations were carried out on a Hitachi (Tokyo, Japan) H-7100 electron microscope with an acceleration voltage of 120 kV at room temperature. The TEM sample was prepared by dropping the sample suspension on a Cu grid and allowed to dry at room temperature. Particle size distribution and the mean size of 50 nanoparticles were assessed on the basis of three TEM images with the assistance of Sigma-Scan Pro software (SPSS IBM, Statistics 20, IBM Corporation, Endicott, NY, USA). Field emission scanning electron microscopy (FESEM) was performed using a JSM-6360LA field emission scanning electron microscope (Philips, Eindhoven, The Netherlands). The powder sample was fixed on a metal stub using carbon tape and then gold-coated using a sputter coater. The sample solutions were analyzed at room temperature for UV-Visible absorption using UV-Vis spectrophotometer (a Lambda 25-Perkin Elmer, Waltham, MA, USA). The absorbance spectra were scanned in the range of 200–800 nm with a 1 nm interval at room temperature. The laser Doppler electrophoresis technique was used to measure the particle electrostatic charge, in which 100 µL of the solution was diluted with 1.5 mL of water and placed into a cuvette of the Zeta sizer-nano instrument (Malvern, UK); the results are expressed as zeta potential (ZP). The measurements were performed at a pH of 7.26 ± 0.13 to mimic physiological pH.

### 3.4. Antimicrobial Assays

The *in vitro* antibacterial ability of the bioformed Ag-NPs was evaluated by using the disc diffusion method using Müeller-Hinton Agar (MHA) with determination of inhibition zones in millimeter (mm), which conform with recommended standards of the National Committee for Clinical Laboratory Standards (NCCLS). *E.coli* (ATCC25922) was used for the antibacterial power assay. Briefly, the sterile paper discs (6 mm) soaked with different concentrations 10, 20, 30, 50, 70 and 90 μg/mL of Ag-NPs and were allow to dry at 37 °C for 24 h in a sterile condition. The bacterial suspension was prepared by making a saline suspension of isolated colonies selected from tryptic soy agar plate, the agar plates were grown for 24 h at 37 °C, on a rotary shaker at 200 rpm. The suspension was adjusted to match the tube of 0.5 McFarland turbidity standard using the spectrophotometer of 600 nm, which equals to 1.5 × 10^8^ colony-forming units (CFU)/mL. The surface of MHA was entirely inoculated using a sterile swab, which immersed in the prepared suspension of bacterium. Finally, the impregnated discs were placed on the inoculated agar and incubated at 37 °C for 24 h. After incubation, the diameter of the growth inhibition zones was measured. *Streptomycin* was used as standard positive control was used as the positive standard in order to control the sensitivity of the bacteria. All tests were replicated three times.

### 3.5. Cytotoxicity Test

HepG2 cells were maintained at 37 °C in a humidified incubator containing 5% CO_2_. Cells were grown in DMEM medium containing 1% penicillin/streptomycin and supplemented with 10% fetal bovine serum (FBS). We performed MTT assays to determine the effect of biosynthesized Ag-NPs on HepG2 cell growth and viability. Briefly, cells were plated at a density of 5 × 10^3^ cells/well in 96-well in final volume of 100 µL of DMEM. After 24 h incubation the medium was removed, and appropriate concentrations of Ag-NPs (0, 0.58, 1.17, 2.34, 4.78 µM) was added to the each wells and incubated for 24 h. After incubation times, 20 μL MTT was added to each well (2 mg/mL) for another 4 h at 37 °C. The formazan product was dissolved in DMSO. The plate was then read on a spectrophotometer (Epoch, Bio Tek, Winooski, VT, USA) at 570 nm.

## 4. Conclusions

We reported a simple green process to synthesize silver nanoparticles by using of *sumac* extract which also acts as a capping agent. The outcomes of this study support the idea of producing silver nanoparticles by the phenolic compounds existing in *sumac* extract. Such a process for synthesizing Ag-NP will offer a benefit for *sumac* and other plant extract containing polyphenols and can potentially participate in producing nanostructures and be converted into high value nanomaterials. The biosynthesized nanoparticle is expected to have potential applications in various biomedical uses such as cancer and antimicrobial therapy.
